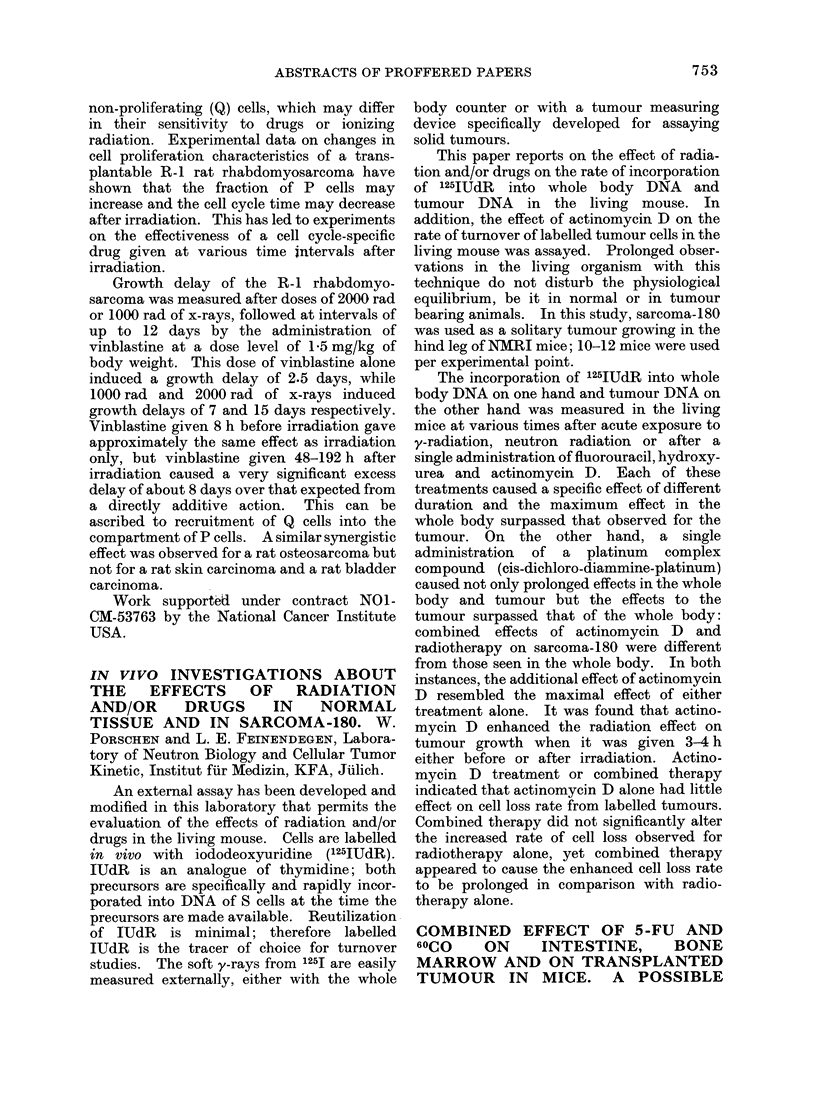# Proceedings: In vivo investigations about the effects of radiation and/or drugs in normal tissue and in sarcoma-180.

**DOI:** 10.1038/bjc.1975.296

**Published:** 1975-12

**Authors:** W. Porschen, L. E. Feinendegen


### *IN VIVO* INVESTIGATIONS ABOUT THE EFFECTS OF RADIATION AND/OR DRUGS IN NORMAL TISSUE AND IN SARCOMA-180.

W. PORSCHEN and L. E. FEINENDEGEN, Laboratory of Neutron Biology and Cellular Tumor Kinetic, Institut für Medizin, KFA, Jülich.

An external assay has been developed and modified in this laboratory that permits the evaluation of the effects of radiation and/or drugs in the living mouse. Cells are labelled *in vivo* with iododeoxyuridine ($^{125}$IUdR). IUdR is an analogue of thymidine; both precursors are specifically and rapidly incorporated into DNA of S cells at the time the precursors are made available. Reutilization of IUdR is minimal; therefore labelled IUdR is the tracer of choice for turnover studies. The soft $\gamma$-rays from $^{125}$I are easily measured externally, either with the whole body counter or with a tumour measuring device specifically developed for assaying solid tumours.

This paper reports on the effect of radiation and/or drugs on the rate of incorporation of $^{125}$IUdR into whole body DNA and tumour DNA in the living mouse. In addition, the effect of actinomycin D on the rate of turnover of labelled tumour cells in the living mouse was assayed. Prolonged observations in the living organism with this technique do not disturb the physiological equilibrium, be it in normal or in tumour bearing animals. In this study, sarcoma-180 was used as a solitary tumour growing in the hind leg of NMRI mice; 10–12 mice were used per experimental point.

The incorporation of $^{125}$IUdR into whole body DNA on one hand and tumour DNA on the other hand was measured in the living mice at various times after acute exposure to $\gamma$-radiation, neutron radiation or after a single administration of fluorouracil, hydroxyurea and actinomycin D. Each of these treatments caused a specific effect of different duration and the maximum effect in the whole body surpassed that observed for the tumour. On the other hand, a single administration of a platinum complex compound (cis-dichloro-diammine-platinum) caused not only prolonged effects in the whole body and tumour but the effects to the tumour surpassed that of the whole body: combined effects of actinomycin D and radiotherapy on sarcoma-180 were different from those seen in the whole body. In both instances, the additional effect of actinomycin D resembled the maximal effect of either treatment alone. It was found that actinomycin D enhanced the radiation effect on tumour growth when it was given 3–4 h either before or after irradiation. Actinomycin D treatment or combined therapy indicated that actinomycin D alone had little effect on cell loss rate from labelled tumours. Combined therapy did not significantly alter the increased rate of cell loss observed for radiotherapy alone, yet combined therapy appeared to cause the enhanced cell loss rate to be prolonged in comparison with radiotherapy alone.